# Addison’s Disease

**Published:** 1880-08

**Authors:** Thos. F. Rochester

**Affiliations:** Professor of Principles and Practice of Medicine, University of Buffalo


BUFFALO

MEDICAL AND SURGICAL JOURNAL.

VOL. XX.—AUGUST, 1880.—No. 1.

### Original Communications.

### ADDISON'S DISEASE.*

BY THOS. F. ROCHESTER, M. D.,

Professor of Principles and Practice of Medicine, University of Buffalo.

SO-CALLED after the eminent English physician, attached to Guy's Hospital, who first recognized and described it in 1849, and subsequently in 1854 published a treatise upon the same, was regarded by him as a distinct, and so to speak, sui generis disorder. This opinion has often been questioned, but is generally endorsed by the medical profession. It may not be out of place to quote the description as originally enunciated by the author.

"The patient gradually becomes languid, weak and indisposed either to bodily or mental exertion, appetite impaired or entirely lost. The white of the eyes becomes pearly, the pulse small and feeble or perhaps small and compressible, the body wastes without presenting the dry shrivelled skin and extreme emaciation usually attendant upon protracted malignant disease; slight pain and uneasiness is from time to time referred to the region

* Read before the Buffalo Medical Association, July 1880.

of the stomach, and there is occasional actual vomiting, which in one instance was both urgent and distressing, and it is by no means uncommon for the patient to manifest symptoms of disturbed cerebral circulation. Notwithstanding these unequivocal signs of feeble circulation, anæmia and general prostration, neither the most diligent inquiry, nor the most careful physical examination, tends to throw the slightest gleam of light upon the precise nature of the patient's malady, nor do we succeed in fixing upon any special lesion as the cause of this gradual and extraordinary change. With more or less of these symptoms we discover most remarkable, and as far as I know, characteristic discoloration taking place in the skin, sufficiently marked, indeed, to have attracted the attention of the patient himself or the patient's friends. The discoloration pervades the whole surface of the body, but is commonly most strongly manifested on the face, neck, superior extremities, penis and scrotum, and in the flexures of the axilla and around the navel. It may be said to present a smoky or dingy appearance or various shades of deep amber or chestnut brown; and in one instance the skin was so universally and so deeply darkened, that but for the features the patient might have been mistaken for a mulatto. If I see a patient who presents this peculiar discoloration of the skin, and with this a certain train and combination of symptoms, a pearly eye, a feeble pulse, a disposition to strongly-marked anæmia and a few other symptoms less constant, I say this is a case in which you will find disorganization of the supra-renal capsules."

The above is taken from the essay of Sam'l Wilks in "Reynold's System of Medicine," but no mention is made of Addison's idea of the exact pathological changes in the capsules, except further on where reference is made to the relative correspondence to the coloration and the capsular degeneration. Wilks describes the structural changes as follows: first, great enlargement deposition of a lardaceous or anyloid material, softening of this, with prominent yellow masses of various sizes surrounded by a translucent semi-fluid albuminous substance, sometimes purulents depots—finally complete yellow granular degeneration and sometimes entire absence of one or both capsules, they and their contents having been destroyed by destructive metamorphosis. These are not his words but a condensation of his description—substantially it corresponds with that of most other writers—who like Addison and Wilks, (the pupil of the former), consider it a specific disease. Some attributing it to tubercle, some to inflammation, some to peculiar blood change induced by spinal or gastric nerve morbid processes, while others hold that the asthenia and some other symptoms are caused by the implication of the great sympathetic nerve by the mechanical pressure of the enlarged supra-renal gland. In short, all confess ignorance as to exact pathology and etiology, and admit the futility of treatment. The speaker is not so presumptuous as to claim that he can throw any more light upon this *dark* subject than his predecessors have done. His object in presenting it this evening being simply to narrate three cases which have come under his observation within a few months, and in two of which there have been much more serious pathological lesions elsewhere than in the supra-renal capsules. Although very characteristic as seen there, but going to prove if they prove anything, that Martineau had reason in asserting "that the malady of Addison should not be considered as a pathological entity." In confirmation of this opinion, it is beyond question, that the general symptoms, including the coloration, have been observed where no disease of the supra-renal bodies was found on post-mortem examination, and on the contrary the disorder has been detected in the supra-renal capsules, when during life, there was no manifestation of signs or symptoms.

Without further remarks the cases will now be briefly presented.

Case First.—Was consulted in my office in November, 1879, by Mrs. O., of Geneseo, a widow, 48 years of age, who had ceased menstruating for more than a year. She thought she was suffering from dyspepsia and liver disorder; she was of medium size, complained of great debility, of breathlessnes, of loss of appetite, of palpitation of the heart, and of constant sounds and roaring in the ears, (anæmic?) but no deafness. Her color was notably dark (sunburned hue), and deeper than elsewhere on the brow, face, neck, hands, nipples, umbilical region and vulva. The sclerotic was clear and pearly, urine normal, uterus free from disease or displacement, pulse 80 and very feeble. The heart sounds were free from murmurs, but very weak. Respiratory sounds good. No spinal tenderness, but pain on percussion over the kidneys, and quite intense when the region was grasped from behind forwards and compressed between the fingers and thumb.

Diagnosis.—Addison's disease; ordered generous diet, passive exercise in the open air; a belladonna plaster over the renal region and prescribed Phosphoric acid ʒi, three times daily, with Quinine gr. i, and ext. nucis, vom. gr. s.

She came again in December, reporting herself stronger and better in every way; continued prescription with addition of ʒii of cod liver oil.

On January 19th, 1880, I received the following letter: "I would prefer not to come to Buffalo just now. For a month, or more, after commencing your treatment, I was entirely free from the constant and most disagreeable roaring in my head, the soreness and pain in the stomach nearly gone and strength and appetite much improved. I regret to say that the first two have returned with the addition of bad circulation, numbness in the body and arms after sleeping. * * * * I fear I have a chronic dyspepsia, which is often aggravated by my anxieties and other mental disturbances."

I did not see her again. In February was telegraphed to visit her, but could not leave home. She died the next day, having been ill six days with pneumonia.

For the post mortem appearances and brief account of her last sickness I am indebted to Dr. W. E. Lauderdale, Jr., of Geneseo: "The whole of the right lung and the lower lobe of the left lung were entirely hepatized; adhesions on right side numerous, some of long standing; heart, normal; stomach, the mucous membrane of the cardiac end was very much thickened, the whole organ was covered with thickened patches; intestines, normal; liver, very pale, size normal; spleen and uterus normal. Kidneys—A section of the left kidney, with the left supra-renal capsule, I sent you last week. The right supra-renal capsule was entirely wanting. Its space was filled with a very thin connective tissue." The fact that one of these bodies has been entirely wanting, is a conspicuous item in the literature of this disease. It is suggested by the speaker, that this may not be the case as often as supposed. The readiness with which it separates from the kidney, on the removal of that organ, and lies concealed in the adjacent fat, may cause it to be overlooked, as was done in case No. 3, until a search was instituted by my suggestion. You have now the capsule from Mrs. O. before you; you will observe that it is enlarged, and fatty, and presents in its centre a dark brown patch, nearly an inch square, composed of pigmentary tissue and granular bodies, such as Wilks describes as forming the last stage of the disease, and such as you will find abundantly shown in the other morbid specimens to be presented. The patient died of an intense pneumonia, which probably had no connection with the capsular disease, but in this, that its fatal issue was hastened by it, and affords an opportunity of seeing the Addisonian disease, at a less advanced stage than usual. It is fair to consider this latter as the foundation of the patient's ill health, but we must not be unmindful of the chronic gastritis and of the possible yellow atrophy of the liver, which was described by Dr. Lauderdale as very pale.

I am indebted to Drs. Fredericks and Macbeth, internes at the Buffalo General Hospital, for the two following reports, having also been present at the autopsy of the latter. I had seen

"Case 2d" three years ago, two or three times at my office, and had then diagnosed Albuminuria.

Case second—Christopher Davies, Englishman, aged 57, a gardener, entered the hospital March 17, 1880. He had been in the hospital several times for short periods during the last two years; had been addicted to excessive use of alcohol for the last twenty years. Although fat, he was feeble and anæmic, pulse slow, regular, large but very compressible; complained of Prurigo, scratching almost constantly; had harrassing cough with copious expectoration; appetite good, but unable to retain much food; urine scanty, high colored, about ten ounces per diem, half its bulk albumen (by tests) and full of hyaline, fatty and granular casts. Exhibited large bronzed spots upon back, chest, arms, legs, scrotum and penis. No signs pointing to Addison's disease had ever been noticed during previous sojourns in the hospital; was delirious at times but never had convulsions; April 14, at 9 A. M., began to show signs of exhaustion and died at 11.30 A. M.

Post-mortem examination 24 hours after death. Liver, normal in size, large bronzed spots on surface.

Spleen atrophied, weight 3½ ounces granular, also bronzed in spots on exterior; heart, hypertrophied, weight 20 ounces; atheroma around mouth of coronary arteries, and in the arch of the aorta.

Kidneys, capsules adherent, left weight ʒi, corticle portion thin, pyramids nearly destroyed, pelvis filled with shot-like calculi; right kidney, weight 2 ounces; other points similar to left.

Supra-renal capsules, left entirely destroyed; right, the accompanying specimen. (Reported by Dr. C. C. Fredericks).

The specimen is now shown, it is enlarged and filled entirely with the yellow granular bodies pathologically characteristic of the last stage of Addison's disease.

Case third.—Thos. Lock, aged 50, native of England, gardener, entered hospital May 7, 1880. Diagnosis, dyspepsia, jaundice, and hypochondriasis. Symptoms on entering, some tenderness over gall bladder; very tender over stomach, which always seems full; does not vomit; fugitive pains over various regions. Dr. Diehl directed tr. iodine over epigastric and hepatic regions, and tonics, pepsin, and 1/4 gr. podophyllin at bedtime. He gradually improved until June 4th, when he had an attack of syncope, after which he was confined to bed. June 5th, Dr. Diehl discovered a tumor, supposed to be mesenteric. June 14th, died suddenly of syncope.

Post-mortem 4 P. M. same day. Stomach greatly distended; pylorus much thickened, with polypoid growth half an inch in diameter and nearly an inch long, springing from it, and extending into the stomach. Peritoneum, and under surface of diaphragm, studded thickly with cancerous or tubercular nodules, from size of a small shot to that of a pea.

Greater omentum, a thick mass also filled with these deposits. This was the tumor diagnosed by Dr. Diehl. Lungs entirely free from disease; supra renal capsules, both diseased. Left atrophied, both contained a dark brown substance. (Reported by Dr. C. A. McBeth).

These remarkable pathological specimens you now see, they are certainly rare and unusual, whether they are cancerous or tubercular has not been determined, but the writer thinks they are of the former character. The supra-renal capsules are almost identical in appearance with that of case second.

Case first was perhaps a good illustration of the Addisonian disorder, but it was not free from complication. The other two, certainly justify the assertion of Martineau already quoted, "that the malady of Addison should not be considered as a pathological entity."

Case No. 3 had no bronzed discoloration, but was cachectic in appearance, with hundreds of points of purpura hæmorrhages about the lower extremities, and yet the supra-renal changes were as marked as possible. Three instances are not enough to draw decided conclusions from, but many authorities are quoted by Flint and others tending to show that where Addison's disease does exist, it is usually complicated with serious disease of other organs, and that while the capsular disorder may be a factory in either a primary or secondary manner in producing a fatal issue, the question of individual pathological identity still remains open.

